# Invasive sinus aspergillosis with mycotic aneurysm of the vertebral artery and subarachnoid hemorrhage – Case report

**DOI:** 10.1016/j.radcr.2021.06.041

**Published:** 2021-07-16

**Authors:** Maruša Mencinger, Tadeja Matos, Katarina Šurlan Popović

**Affiliations:** a Clinical Institute of Radiology, University Medical Centre Ljubljana, Zaloška 7, 1000 Ljubljana, Slovenia; b Institute of Microbiology and Immunology, Faculty of Medicine, University of Ljubljana, Ljubljana, Slovenia; c Faculty of Medicine Ljubljana, University of Ljubljana, Ljubljana, Slovenia

**Keywords:** Aspergillosis, Sinusitis, Invasive sinus aspergillosis, Mycotic aneurysm, Perineural spread, Subarachnoid hemorrhage

## Abstract

Invasive sinus aspergillosis is a rare life-threatening condition usually found in immunocompromised patients. The fungus spreads from paranasal sinuses into the central nervous system by direct extension or through blood vessels. Perineural spread is an uncommon mechanism of spread in invasive aspergillosis. A mycotic aneurysm is a dangerous complication of invasive sinus aspergillosis because of its insidious development and is often diagnosed only post-mortem after causing fatal intracranial hemorrhage. Intracranial vascular complications of invasive sinus aspergillosis require prompt recognition and treatment and should always be considered when a diagnosis of CNS aspergillosis is made. We present a case of invasive sinus aspergillosis in an apparently immunocompetent patient that manifested with a brain abscess, perineural spread of the infection, and mycotic aneurysm of the vertebral artery with subsequent rupture and fatal subarachnoid hemorrhage. This case highlights the possibility of perineural spread and hemorrhagic complications in invasive cerebral aspergillosis.

## Introduction

*Aspergillus* species are among the most common causes of fungal sinusitis around the world [1]. Fungal sinusitis presents with various clinical syndromes, ranging from benign sinonasal colonization to life-threatening invasive intracranial disease. Invasive forms of the disease with intracranial spread usually occur in immunocompromised patients, although case reports of cerebral aspergillosis in immunocompetent individuals exist [1]. Intracranial sinus aspergillosis complications include brain abscess, meningitis, vasculitis, and mycotic aneurysm [2]. Perineural spread of aspergillosis is sporadic, with not many reports in the literature.

### Case presentation

A 66-year-old man presented to the neurology emergency unit with a 1-month history of progressive gait instability, cognitive decline, and double vision, accompanied by weight loss for one year. He also had a 1-year history of symptoms of left-sided trigeminal neuralgia. His past medical history included insomnia and episodes of diarrhea with melena of unknown etiology. Neurological examination on admission revealed moderate cognitive impairment (MMSE score was 18 out of 30 points), slightly constricted left pupil, double vision in all directions of gaze, fasciculations in the lower half of the face, and irregular rest tremor in all 4 limbs. Initial laboratory tests showed minimal elevation of C-reactive protein and leukocytosis with relative lymphopenia (relative lymphocyte count 17%). Serologic tests for human immunodeficiency virus (HIV), *Treponema pallidum*, and *Borrelia burgdorferi* were negative.

**Fig. 1:**
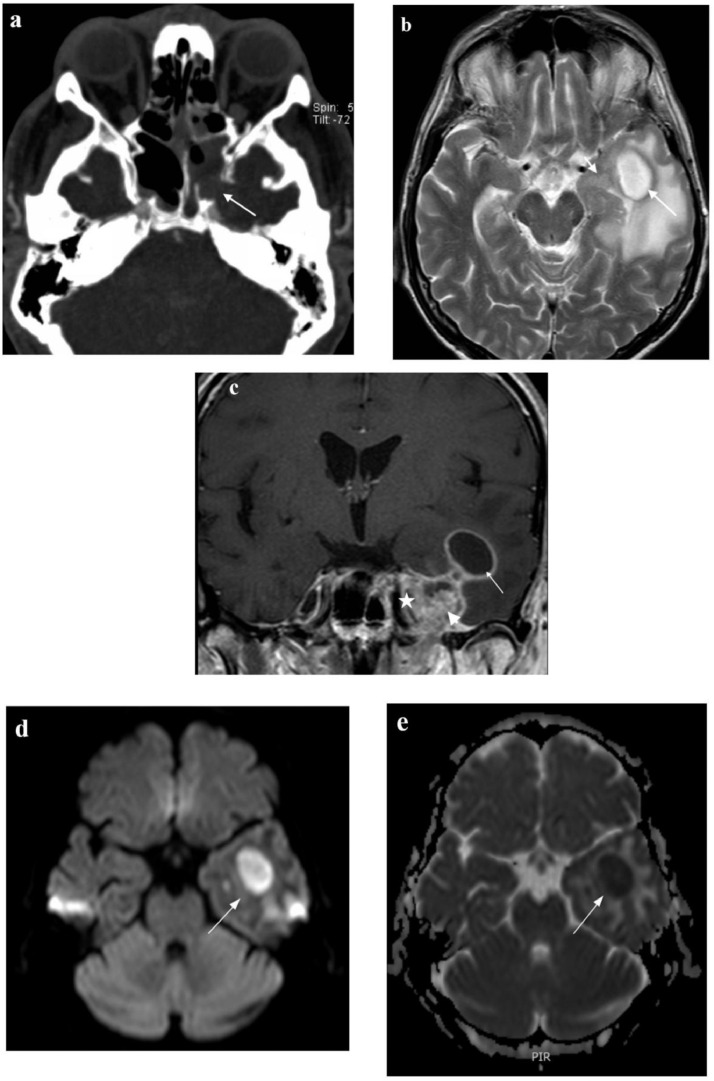
(Imaging on admission). (A) Head CT in axial plane (bone window) shows thickened mucosa and a defect in the lateral wall (arrow) of the sphenoidal sinus. (B) The cystic part of the temporal lobe lesion has a T2 hypointense rim (long arrow), and the solid part has a a T2 hypointense signal (short arrow). (C) Corresponding T1 obtained after gadolinium administration showed rim enhancement of the cystic part (arrow) and heterogeneous enhancement of the solid part (arrow head), also affecting the cavernous sinus (asterix). (D) DWI sequence shows a hyperintense lesion with a slightly hypointense center (arrow). (E) There is marked restriction of diffusion on ADC maps in the cystic part (arrow).

Non-contrast head computed tomography (CT) performed in the emergency department demonstrated a hypodense lesion in the left temporal lobe with surrounding vasogenic edema. The local mass effect caused gyri effacement, compression of the left lateral ventricle, and mild rightward midline shift. Thickened mucosa and partial bone destruction of the lateral wall of the left sphenoid sinus was seen (Fig. 1A). A gadolinium contrast-enhanced MRI of the brain and face revealed a partially cystic tumorous lesion in the left temporal lobe surrounded by vasogenic edema. The lesion's cystic part had a T2 hypointense rim, enhancing after contrast agent (Fig. 1B), and demonstrated central restriction of diffusion on diffusion-weighted imaging (DWI) and on apparent diffusion coefficient (ADC) maps (Figs. 1D and 1E). The solid part of the tumor was hypointense on T2-weighted images, affecting the left cavernous sinus and lateral wall of the sphenoid sinus. The solid part of the tumor enhanced heterogeneously with contrast agent (Fig. 1C).

**Fig. 2:**
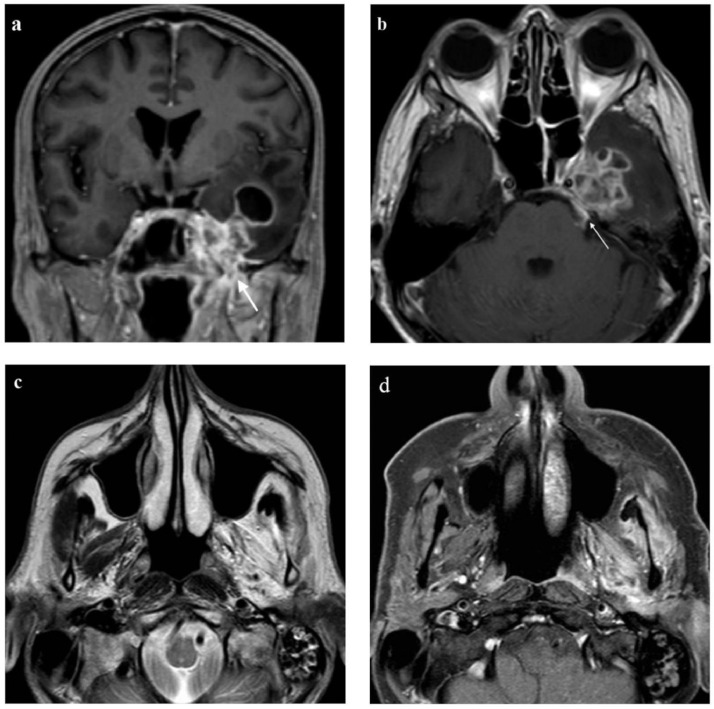
(Imaging on admission). (A) Contrast enhanced T1-weighted image in coronal plane reveals thickening and enhancement of the third branch of the trigeminal nerve in left foramen ovale (arrow). (B) Contrast enhanced T1-weighted image in axial plane shows thickening and enhancement of the cisternal part of the left trigeminal nerve (arrow). (C) Muscles of the left masticator space demonstrate a T2 hyperintense signal and (D) enhance after contrast agent due to denervation.

Contrast-enhanced T1-weighted images also revealed thickening and enhancement of the adjacent dura, the third branch of the trigeminal nerve in the left foramen ovale, the first and the second branch of the trigeminal nerve in the left cavernous sinus and cisternal part of the left trigeminal nerve (Figs. 2A and 2B). Muscles of the left masticator space had a T2 hyperintense signal and enhanced with contrast agent (Figs. 2C and 2D).

The primary differential diagnosis included an invasive fungal infection, probably aspergillosis with brain abscess, perineural spread, and mastication muscle denervation, given the CT and MRI findings.

The patient was started on dexamethasone and combination antimicrobial therapy consisting of cefepime, metronidazole, and amphotericin B. Surgical debridement of the left sphenoid sinus was performed, and histopathological examination revealed chronic ulcerative inflammation of the sinus mucosa caused by *Aspergillus fumigatus*. Additionally, *Raoultella ornithinolytica* and *Cutibacterium acnes* were also isolated in the specimen. Blood tests showed increased levels of nonspecific fungal biomarker 1,3-β-d-Glucan (BDG) and a borderline positive value of galactomannan antigen. Susceptibility testing of the fungal isolates showed in-vitro sensitivity to voriconazole and amphotericin B, and therapy with amphotericin B was subsequently replaced by voriconazole. Following the sinus debridement and antimicrobial therapy, the patient began to improve and reported less neuralgia-associated pain.

During hospitalization, additional blood tests were performed to identify a possible immunodeficiency that could explain the invasiveness of the infection. The only abnormality found was a low level of gamma globulins.

**Fig. 3:**
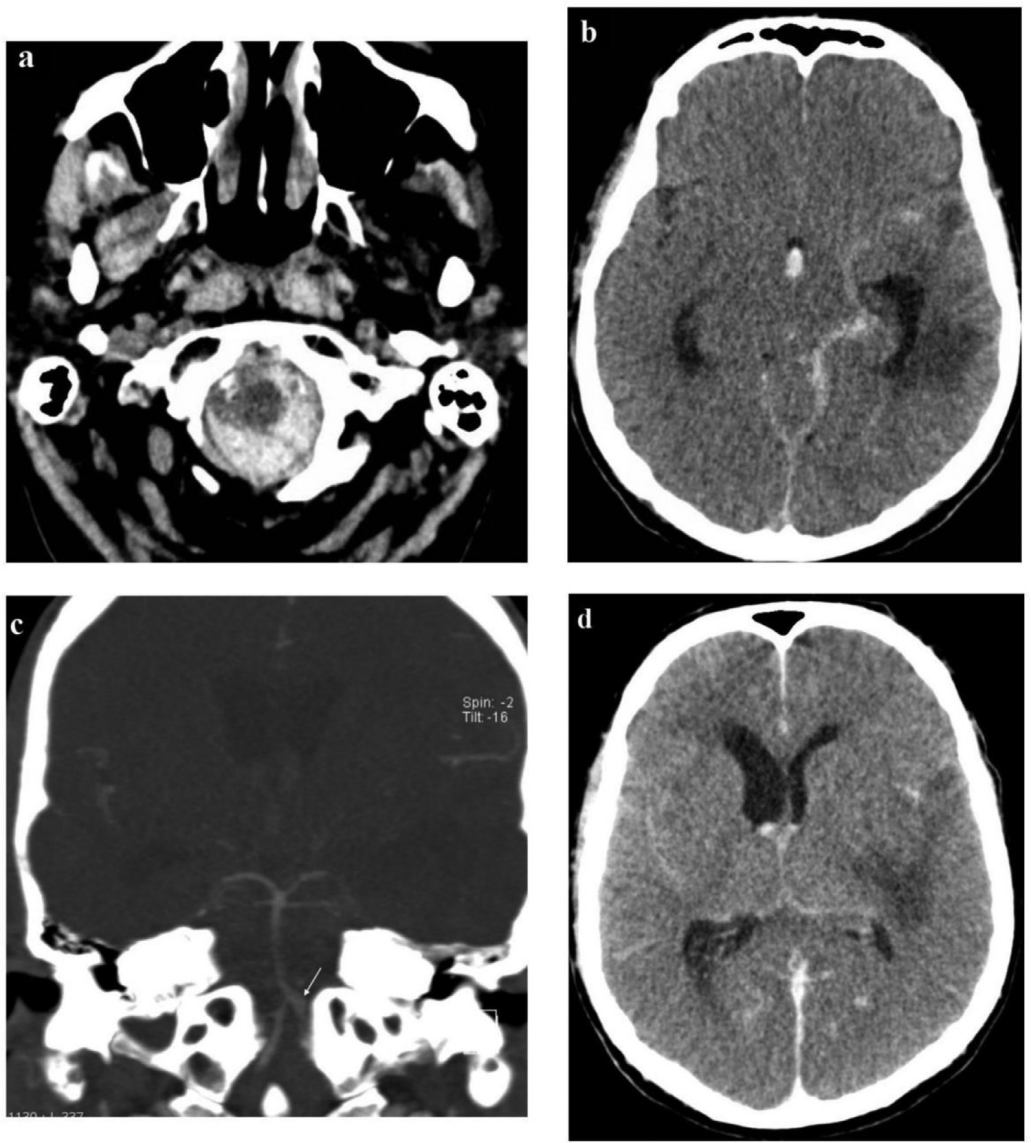
(Hospital day 16). (A) Head CT in axial plane shows subarachnoid hemorrhage in foramen magnum. (B) Subarachnoid hemorrhage in the prepontine and interpeduncular cisterns, left ambient cistern, and left sylvian fissure. (C) CTA of intracranial arteries reveals a fusiform dilatation of the left vertebral artery (arrow). (D) Repeated head CT 12 hours after resuscitation shows progression of subarachnoid hemorrhage with blood in the cerebral sulci and in the third and fourth cerebral ventricle, along with development of diffuse cerebral edema.

On the 16th day of hospitalization, the patient was found unresponsive in his bed with no palpable pulse and no spontaneous respiration. Immediately following resuscitation, head CT was performed, which revealed subarachnoid hemorrhage (SAH) in the foramen magnum, prepontine, and interpeduncular cisterns, left ambient cistern and left Sylvian fissure, with brain edema in the left temporal lobe (Figs. 3A and 3B). Computed tomography angiography (CTA) showed a fusiform dilatation of the left vertebral artery with a 4 mm diameter, extending over a distance of 7 mm and terminating at the vertebrobasilar junction (Fig. 3C). Repeated head CT 12 hours after resuscitation showed SAH progression with blood in the cerebral sulci and in the third and fourth cerebral ventricle, left-sided uncal herniation, and development of diffuse cerebral edema (Fig. 3D). Compression of the left cerebral ventricle and dilatation of the right ventricle prompted the insertion of external ventricular drain (EVD).

Despite interventions, the patient's condition continued to deteriorate, spontaneous breathing ceased, his pupils remained fixed and dilated, and brainstem reflexes could not be elicited. Given the poor prognosis at this stage, a decision was made to limit therapy after consultation with the patient's family. The patient died on day 18 after admission.

Brain autopsy revealed generalized cerebral edema with tonsillar and transtentorial herniation. Moderate to severe diffuse hypoxic brain damage was observed. There was prominent subarachnoid hemorrhage predominantly in the basal cisterns with extension into the ventricular system. Additionally, localized *Aspergillus* meningitis of the brain base with invasive aspergillosis of the left hippocampus was observed. The cause of death was a ruptured mycotic aneurysm of the left vertebral artery, caused by *Aspergillus spp*.

## Discussion

Invasive aspergillosis of the central nervous system (CNS) can manifest as a brain abscess, meningitis, or in the form of vascular complications. The fungus usually spreads from the sinuses by bone erosion or by an invasion of blood vessels [1]; reports of perineural spread of *Aspergillus* infections are rare [3], [4], [5], [6], [7]. In the 5 cases we found in the literature, perineural *Aspergillus* invasion involved the maxillary branch of the trigeminal nerve in 2 cases [4,7], the optic nerve in 2 cases [5,6], and the facial nerve in 1 case [3]. Interestingly, 4 out of the 5 patients with perineural spread were immunocompetent. In our case, the perineural invasion was more extensive as it involved all 3 branches of the trigeminal nerve. MRI signs of perineural spread include thickening and contrast enhancement of cranial nerves, while an indirect indication of perineural infiltration is muscle denervation, demonstrated by hyperintense T2 signal and contrast enhancement of muscles [4,8]. In our case, we observed signs of denervation of the left mastication muscles, which showed high signal intensity on T2 and enhancement after contrast agent. These MRI changes are typically seen in the acute phase of muscle denervation, which most authors define as a duration of 1 month or less, and are considered to be the result of edema-like changes in the denervated muscle [8]. Acute denervation of mastication muscles in our case developed due to fungal perineural invasion of the mandibular branch of the trigeminal nerve, which innervates these muscles. Up to 40% of patients with perineural infiltration are asymptomatic [9]. In the case of our patient, the perineural infiltration and subsequent muscle denervation clinically manifested as trigeminal neuralgia and fasciculations on examination.

CT scan is usually nonspecific in CNS aspergillosis, and MR imaging is essential for diagnosis [2]. CNS aspergillosis lesions can be divided into 2 types: meningeal lesions and parenchymal lesions. Meningeal invasion produces nonspecific MRI features with dural enhancement and thickening, which was also observed in our case. Typical MRI findings in parenchymal lesions include: hypointense signal on T2 images, hypo- to iso-intense signal on T1 images, and restricted diffusion on DWI sequences [10], [11], [12].

Enhancement patterns in brain aspergillosis vary. The absence of contrast enhancement or weak peripheral ring enhancement is deemed characteristic of *Aspergillus* lesions [10]. The lack of contrast enhancement is thought to result from an inadequate inflammatory response due to immunosuppression, although cases of well-defined ring enhancement in immunocompromised patients exist [2,12]. Nevertheless, several researchers report bright, homogeneous enhancement or ring enhancement, generally in less severely immunocompromised patients [11]. Therefore, the absence of contrast enhancement is not a reliable sign of CNS aspergillosis, and DWI imaging is considered the most sensitive modality for timely diagnosis [2,12]. DWI in cerebral aspergillosis typically shows hyperintense lesions with reduced ADC values, indicating restricted diffusion. Another possible DWI finding is a target-like lesion with hypointense center, surrounded by hyperintense ring [12]. In our case, we saw a similar pattern of a target-like lesion on DWI with hypointensity in the very center of the lesion and the rest of the lesion showing a hyperintense signal.

In our case, the lesion in the temporal lobe exhibited a heterogeneous structure with a cystic component and a solid component. The cystic part showed typical MRI signs of aspergillosis with T1 hypointensity and central restriction of diffusion on DWI. Additionally, a hypointense ring on T2 images was observed. This finding in cerebral aspergillosis is attributed to ferromagnetic elements in peripheral fungal structures [10]. Contrast enhancement was present in both parts of the lesion; the cystic part demonstrated ring enhancement, while the solid part showed heterogeneous enhancement. Perhaps the presence of enhancement can be explained by a relatively preserved immune response in our patient, who had no known chronic illness or immunodeficiency apart from newly discovered hypogammaglobulinemia.

An essential clue to a possible fungal cause of brain lesions is sinus involvement. Fungal sinusitis results in characteristic low T2 hyperintensity and mucosal thickening [10]. In invasive fungal sinusitis, the changes in sinonasal mucosa can be subtle, and careful examination of the surrounding spaces is important for detecting soft-tissue infiltration [1]. Signs of extra sinus invasion are retroantral soft tissue thickening and obliteration of periantral fat, while bone erosion of the sinus wall is best seen on a CT scan [4]. Bone erosion can sometimes be minimal or absent, because the fungi may extend beyond the sinuses via perivascular channels [13], [14], [15].

*Aspergillus* species are angioinvasive; they produce elastase enzyme, which digests elastin in the internal lamina of blood vessels, generating an inflammatory vascular reaction [2]. The weakening of the vessel wall leads to aneurysm formation and its subsequent rupture with subarachnoid hemorrhage. Other vascular complications of invasive intracranial aspergillosis include thrombosis, vasculitis, and fungal embolisms [2]. Intracranial aneurysms as a complication of invasive fungal sinusitis are rare [16]. Most occur in the internal carotid artery because of its proximity to sinuses, as the fungus spreads intracranially by direct invasion of neighboring structures [17]. Less commonly, a fungal aneurysm develops in the arteries of the posterior circulation [2,18]. In our case, the patient developed a fungal aneurysm of the vertebral artery, which is a sporadic condition – only a handful of reports exist in the literature [18], [19], [20].

Kenneth et al. analyzed all cases of infectious aneurysms treated in their institute from 1976 to 2003 and found 1 case of vertebral artery aneurysm caused by *Aspergillus* meningitis [18]. The patient died from SAH, and the aneurysm was diagnosed at autopsy. Other details of the case, including the patient's immune status, were not available.

Partridge and Chin reported a case of vertebral artery aneurysm in a young immunocompetent patient with aspergilloma of the left temporal lobe [19]. The infection originated in the sphenoid and ethmoidal sinuses and spread to the orbit and intracranially through the sphenoid bone. Despite surgical excision and antifungal therapy, the patient died from aneurysmal rupture and subsequent SAH. The clinical course was similar to our case, with no immune deficiency having been found in any of the patients. Interestingly, the aneurysm developed in a similar rapid manner as in our case, although no specific imaging was performed that could perhaps reveal an aneurysm beforehand.

In a third case, Piotrowski et al. described a patient with *Aspergillus* aneurysm of the vertebral artery with rupture and SAH following a neurosurgical procedure [20]. The patient was immunocompromised due to steroid treatment after the surgery.

In our patient, vertebral artery aneurysm most likely developed due to contiguous invasion of the fungus from the temporal lobe aspergilloma and the infected meninges on the brain base. On admission, the differential diagnosis did not warrant specific neurovascular imaging, so the time course of the aneurysm development is unknown.

Mortality in patients with intracranial fungal aneurysms is extremely high [17,21], in part due to the rapid development and rupture of aneurysms. Difficulty in diagnosing CNS aspergillosis and the immunocompromised state of these patients are additional causes of poor prognosis. Successful treatment of *Aspergillus* aneurysms is rare, with few reports in the literature [22,23].

Invasive intracranial aspergillosis is more common in immunocompromised individuals than immunocompetent ones. Our patient appeared to be immunocompetent at first, except for low relative lymphocyte count, commonly considered a marker of stress response in infection [24]. Diagnostic workup for identifying the cause of the patient's invasive infection did not reveal any immune deficiency apart from hypogammaglobulinemia. It is unlikely that hypogammaglobulinemia affected the disease course, as the main defense against fungal infections is cellular immunity [25]. There may be several causes for the aggressive nature of the patient's infection. Firstly, despite high in-vitro sensitivity to voriconazole, the actual tissue penetration may not have been optimal. Furthermore, voriconazole metabolism and plasma concentrations, which can be highly individual [26], were not measured in our patient. Lastly, corticosteroid therapy, intended to reduce cerebral edema, can simultaneously weaken the phagocytic activity of immune cells and in our case probably contributed to the spread of the infection [27].

## Conclusion

Signs and symptoms of invasive sinus aspergillosis may be subtle and nonspecific, so the clinical suspicion must be high, especially in immunocompromised patients. MRI signs of invasive sinus aspergillosis include destruction of the sinus wall, thickening of periantral soft tissues, subtle meningeal thickening and enhancement, and brain parenchymal lesions with restricted diffusion. Perineural invasion is a rare finding in invasive sinus aspergillosis and may be accompanied by muscle denervation, demonstrated by hyperintense T2 signal and contrast enhancement of muscles. Mycotic aneurysm caused by direct extension of invasive sphenoidal aspergillosis is a rare but life-threatening complication. The almost uniformly fatal outcome of intracranial fungal aneurysms implies that early detection of vascular complications of CNS aspergillosis is vital. As soon as CNS aspergillosis is diagnosed, potential vascular involvement should be considered and appropriate diagnostic techniques performed, which may improve these patients' outcomes.
